# Diagnostic Intervals in Children, Adolescents and Young Adults with Primary Bone Sarcoma: A Systematic Review

**DOI:** 10.3390/cancers18162590

**Published:** 2026-08-12

**Authors:** Mathilde F. Køtter, Maya E. R. Schambye, Simon Espensen, Ninna Aggerholm-Pedersen, Daniel T. H. Dybdal, Jesper S. Brok, Lisa L. Hjalgrim

**Affiliations:** 1 Pediatric Oncology Research Laboratory, Department of Pediatrics and Adolescents Medicine, Copenhagen University Hospital–Rigshospitalet, 2100 Copenhagen, Denmark; mathilde.frost.koetter@regionh.dk (M.F.K.); maya.elena.ramirez.schambye.01@regionh.dk (M.E.R.S.); simon.espensen@regionh.dk (S.E.); daniel.thor.halberg.dybdal@regionh.dk (D.T.H.D.); 2 Department of Oncology, Aarhus University Hospital, 8200 Aarhus, Denmark; ninnpede@rm.dk; 3 Childhood Cancer Research Group, Danish Cancer Institute, Danish Cancer Society, 2100 Copenhagen, Denmark; 4 Department of Pediactrics and Adolescent Medicine, Copenhagen University Hospital–Rigshospitalet, 2100 Copenhagen, Denmark; jesper.sune.brok@regionh.dk

**Keywords:** bone sarcoma, systematic review, diagnostic interval, children, adolescents, young adults

## Abstract

**Background/Objectives**: Survival in children, adolescents and young adults (CAYA) with bone sarcoma remains inferior to that of many other early-life cancers. Early recognition and treatment initiation may reduce the risk of presenting with advanced disease. We aimed to synthesize evidence on five predefined diagnostic intervals, separately synthesize pathway-specific intervals, and identify factors associated with diagnostic interval duration. **Methods**: A systematic review was conducted according to PRISMA guidelines. We included studies reporting the duration of a diagnostic interval in at least five patients aged 0–39 years with primary bone sarcomas, including osteosarcoma, Ewing sarcoma, and less common histological subtypes. MEDLINE, Embase, the Cochrane Central Register of Controlled Trials (CENTRAL), Web of Science, and Scopus were searched for studies published between 2000 and 2025. Risk of bias was assessed using a custom domain-based tool. **Results**: Twenty-nine studies including 9271 patients from high-income countries met the inclusion criteria. Substantial heterogeneity in study design, populations, and interval reporting precluded meta-analysis. Reported median intervals were patient intervals of 13–84 days, diagnostic intervals of 15–123 days, total diagnostic intervals of 28–150 days, treatment intervals of 7–24 days, and total intervals of 66–88 days. Older age within the CAYA population and axial tumor location were the factors most consistently associated with longer diagnostic intervals. No consistent association between diagnostic interval duration and survival was identified. Pathway-specific intervals provided additional insight into different components of the diagnostic pathway, particularly after initial healthcare contact, although evidence remained limited. **Conclusions**: Diagnostic interval durations varied substantially across studies, reflecting methodological heterogeneity and differences in diagnostic pathways. Standardized prospective studies are needed to strengthen the evidence base.

## Simple Summary

Children, adolescents, and young adults with primary bone sarcoma may experience different diagnostic pathways and healthcare-seeking patterns than older adults. However, evidence on diagnostic intervals in this population remains limited. Delayed diagnosis of primary bone sarcomas may result in more advanced disease at presentation. Symptoms are often non-specific and difficult to recognize, which may contribute to prolonged diagnostic intervals. This systematic review synthesized evidence on diagnostic intervals in children, adolescents, and young adults with primary bone sarcomas, including osteosarcoma, Ewing sarcoma, and less common histological subtypes, and explored factors associated with diagnostic interval duration. Diagnostic intervals varied widely across studies, with longer intervals observed among older patients and in those with centrally located tumors. Pathway-specific intervals provided additional insight into the diagnostic process, particularly after initial healthcare contact. No consistent association between diagnostic interval duration and survival was identified. Interpretation of the findings was limited by methodological heterogeneity and variation in diagnostic interval definitions and reporting. These findings highlight the need for more consistent reporting of diagnostic intervals and diagnostic pathways to improve understanding of diagnostic timeliness in children, adolescents, and young adults with primary bone sarcoma.

## 1. Introduction

Bone sarcomas are rare malignant tumors that account for approximately 20% of cancer-related mortality among children, adolescents, and young adults (CAYA; 0–39 years) despite representing only a small fraction of cancers in this age group [1,2,3,4]. In contrast to many other childhood and young adult malignancies, survival has shown minimal improvement over recent decades [3,4,5]. Five-year survival is approximately 70% among children but remains closer to 50% among adolescents and young adults [5,6,7].

Older age, metastatic disease at diagnosis, and axial tumor location are among the strongest adverse prognostic factors [8], with five-year survival falling to 15–35% for patients presenting with metastatic disease [2,5,7]. Because prognosis is closely related to tumor size, metastatic burden, and the feasibility of complete surgical resection [5,7,9,10,11], reducing unnecessary delays before diagnosis and treatment remains an important clinical objective [12].

Achieving timely diagnosis is, however, challenging. Bone sarcomas are rare and typically present with non-specific symptoms such as localized or nocturnal pain or swelling that frequently resemble common benign musculoskeletal conditions, including sports injuries, rheumatological diseases, and growing pains [12]. These diagnostic challenges may be especially pronounced in adolescents and young adults, where developmental, behavioral, and healthcare-seeking factors may further complicate symptom appraisal and clinical assessment [13,14,15,16,17]. Accordingly, patients with bone sarcoma have been shown to experience longer diagnostic intervals than patients with many other pediatric cancers [18,19].

Previous systematic reviews have examined diagnostic intervals across pediatric cancers [18,19] or within broader sarcoma populations that included adults of all ages [20,21]. Consequently, evidence specific to CAYA with primary bone sarcoma remains limited. Given the distinct clinical and healthcare context of CAYA with primary bone sarcoma [22,23], a dedicated synthesis in this population is warranted.

However, meaningful synthesis and comparison across studies are complicated by variation in diagnostic interval definitions and reporting. To facilitate more consistent reporting, Olesen et al. proposed the Anderson Model [24], which was later formalized in the Aarhus Statement to provide standardized definitions of diagnostic intervals [25].

**Figure 1:**
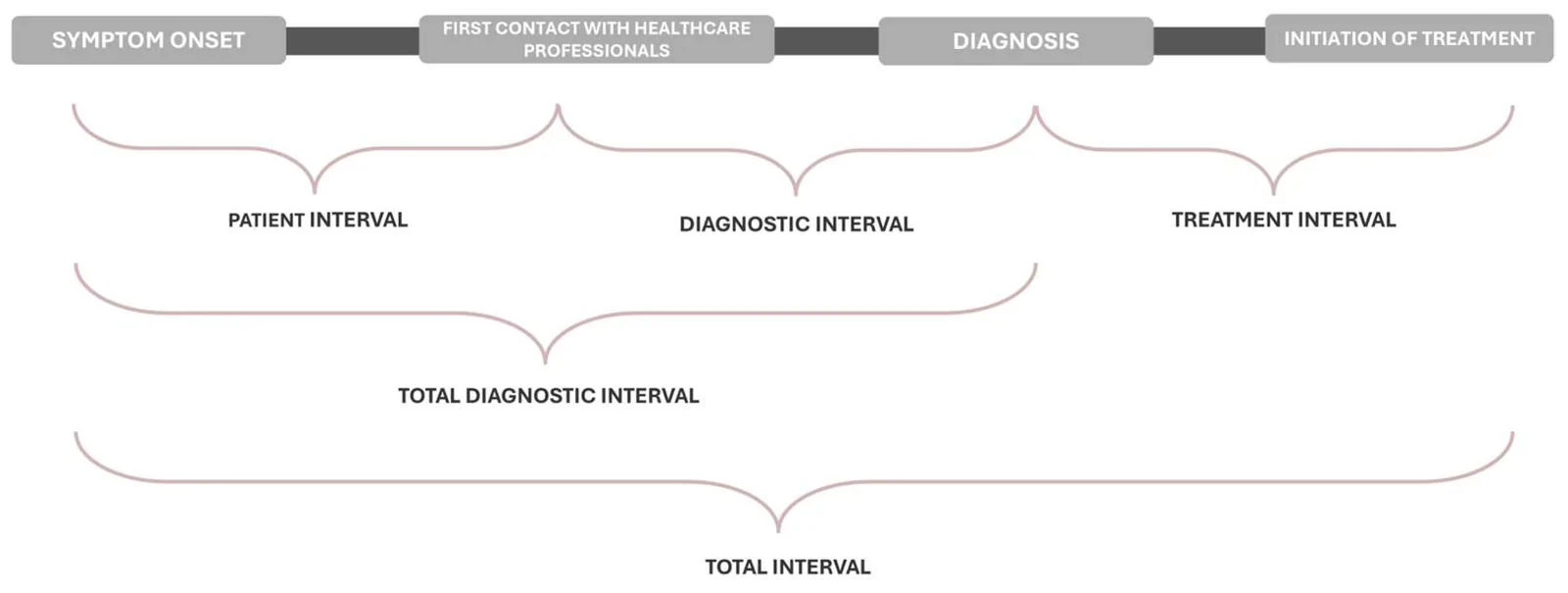
Selected Diagnostic Intervals Based on the Anderson Model [24].

The objective of this systematic review was to synthesize evidence on diagnostic intervals in CAYA with primary bone sarcoma. Specifically, we quantified five predefined diagnostic intervals based on the Aarhus Statement (Figure 1 and Table 1), synthesized additional pathway-specific intervals, and identified factors associated with diagnostic interval duration. By combining predefined diagnostic intervals with pathway-specific intervals, this review aimed to improve comparability across studies while providing a more comprehensive characterization of the diagnostic pathway.

## 2. Materials and Methods

### 2.1. Literature Search

We conducted a systematic review following the guidelines outlined in the Preferred Reporting Items for Systematic Reviews and Meta-Analyses (PRISMA) statement [26] (see Supplementary Material). The review protocol was registered in PROSPERO with ID CRD420250650464 [27].

The search strategy was developed with assistance from an information specialist. The search strategy combined relevant Medical Subject Headings terms and keywords from the relevant literature, covering primary bone sarcomas, diagnostic and treatment intervals, diagnostic and treatment delay, time to diagnosis, time to treatment, and early diagnosis or referral. The complete search strings are provided in Supplementary Table S1.

We searched the electronic databases Embase (Ovid), MEDLINE, the Cochrane Central Register of Controlled Trials (CENTRAL), Web of Science, and Scopus for studies published between 1 January 2000 and 11 February 2025, in English (full text). We also conducted supplementary searches, including forward citation searches of the reference lists of included studies and relevant reviews, to identify additional eligible studies.

### 2.2. Selection Criteria

Studies presenting original data were eligible for inclusion if they met both of the following criteria:

1. Reported on a minimum of five patients aged 0–39 years with a primary diagnosis of bone sarcoma, including osteosarcoma, Ewing sarcoma, and chondrosarcoma, or studies including broader cohorts of primary bone sarcomas or malignant bone tumors;
2. Reported the duration of at least one interval along the diagnostic pathway from symptom onset to treatment initiation.

The upper age limit of 39 years was selected a priori in accordance with the National Cancer Institute definition of adolescents and young adults (15–39 years), while including children (0–14 years) to capture the full CAYA population [28]. Studies of broader cancer cohorts or wider age ranges were included only if they provided detailed subgroup analyses for study participants with bone sarcoma aged 0–39 years. Studies with subgroups of fewer than five study participants were excluded.

Studies were excluded if they included study participants with known sarcoma predisposition syndromes or secondary malignancies or focused exclusively on diagnosis via genetic testing. Studies reporting intervals confined to selected subgroups (e.g., misdiagnosed patients) were excluded. Case reports were excluded to limit selection bias. Studies lacking clear definitions of diagnostic interval start and end points were excluded.

Only studies from high-income countries (HICs) were included, because differences in healthcare system organization, access to diagnostic services, and referral pathways compared with low- and middle-income countries (LMICs) were considered likely to influence diagnostic pathways and interval estimates. HICs and LMICs were identified according to the World Bank’s 2024–2025 income classification [29].

In cases where multiple studies reported data from overlapping populations, the study with the most comprehensive dataset or most relevant outcomes was included to avoid double-counting participants. Overlap was assessed based on study site, data collection period, and author group. When potential overlap was suspected but could not be confirmed, this was noted in the tables.

The inclusion and exclusion criteria are detailed in Supplementary Table S2.

### 2.3. Diagnostic Interval Framework

A modified version of the Aarhus Statement definitions of diagnostic intervals was applied to assess the period prior to treatment initiation [25]. Five predefined diagnostic intervals were examined: (i) the patient interval, (ii) the diagnostic interval, (iii) the total diagnostic interval, (iv) the treatment interval, and (v) the total interval (Figure 1). Additional reported intervals describing specific components of the diagnostic pathway that did not correspond to one of the predefined diagnostic intervals were classified as pathway-specific intervals.

### 2.4. Literature Screening

The literature identified through the search strategy was imported into the web-based software Covidence (Veritas Health Innovation, Melbourne, Australia) [30], and duplicates were removed. Study selection was carried out by four reviewers (M.F.K., D.T.H.D., M.E.R.S., S.E.). Each record was independently screened by two reviewers in a two-stage process: title and abstract screening followed by full-text review. Disagreements between reviewers were resolved either through reaching consensus or by consultation with a third reviewer (D.T.H.D., L.L.H.).

### 2.5. Risk of Bias Assessment

No existing risk-of-bias tool was identified that was specifically designed to assess studies of diagnostic intervals. Therefore, a custom domain-based risk-of-bias assessment tool was developed a priori to evaluate methodological aspects considered most likely to influence the validity, comparability, and interpretation of diagnostic interval estimates. The five assessment domains were selected to capture population representativeness and reporting; definition and measurement of diagnostic intervals; completeness of interval data; transparency and handling of time data; and consideration of bias and confounding. The assessment domains were informed by methodological recommendations from the Aarhus Statement, the Quality Assessment of Diagnostic Accuracy Studies (QUADAS-2), and Reporting studies on time to diagnosis (REST) [25,31,32]. The assessment domains are summarized in Supplementary Table S3. Studies were rated as having low, moderate, or high risk of bias.

The assessment was performed by one reviewer (M.F.K.) and independently reviewed by a second (M.E.R.S.). Any discrepancies in judgements of risk of bias were resolved through discussion or by consultation with L.L.H.

### 2.6. Data Extraction

Data were extracted by one reviewer (M.F.K.) using a predefined extraction form and subsequently checked independently for accuracy by a second reviewer (M.E.R.S.). For each included study, we collected information on study characteristics, population characteristics, and definitions and units of reported diagnostic intervals, which were converted to days to ensure consistency across studies. Definitions of reported interval start and end points were extracted to classify reported intervals according to the predefined diagnostic intervals or as pathway-specific intervals. Because of variation in study design, interval reporting and outcome definitions, a meta-analysis was not feasible. Results were synthesized narratively using the five predefined diagnostic intervals as the primary framework for analysis. In addition, pathway-specific intervals, healthcare entry points, factors associated with diagnostic interval duration, and reported associations with survival were synthesized separately. The extraction forms are available in the Supplementary Material (Supplementary Tables S4 and S5).

### 2.7. Data Visualization

The predefined diagnostic intervals were visualized as boxplots using R version 4.4.1 (R Foundation for Statistical Computing, Vienna, Austria). Only studies reporting a median interval were included. When available, the interquartile ranges (Q1–Q3) defined the box, and the median was shown with a bold line and label. Whiskers were drawn only when a minimum and maximum interval were reported; otherwise, boxes were displayed without whiskers. Likewise, whiskers were displayed without boxes if no interquartile ranges were reported. Axes were log-scaled when all plotted values were >0 after applying a small positive floor to non-positive values.

Pathway-specific intervals were not visualized because differences in interval definitions and reporting precluded meaningful graphical comparison.

## 3. Results

### 3.1. Study Selection

**Figure 2:**
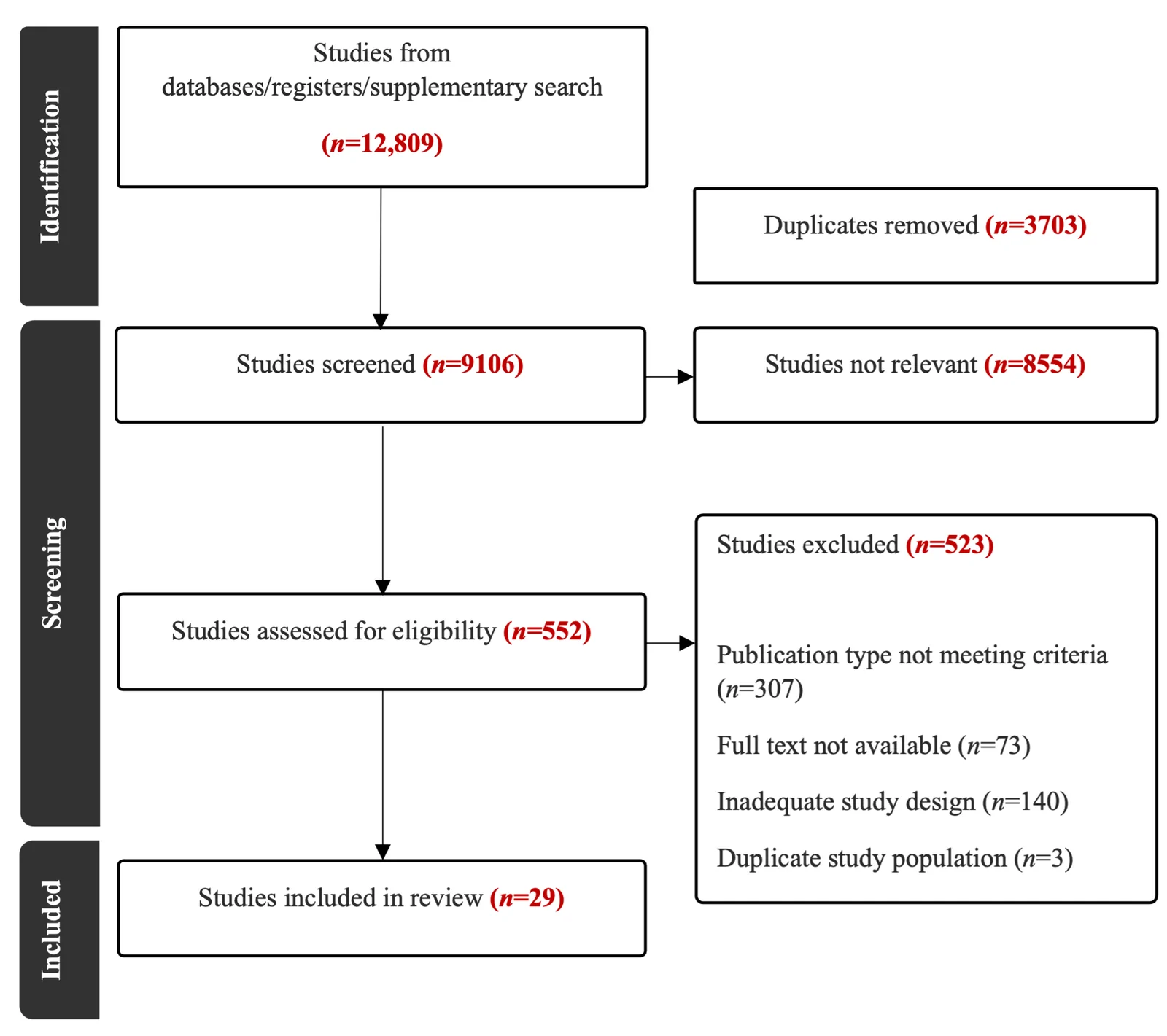
Preferred Reporting Items for Systematic Reviews and Metaanalysis (PRISMA) Diagram.

A total of 12,794 records were identified using database searching. Additionally, reference list screening yielded 14 further studies, and supplementary searching identified one additional study. In total, 12,809 records were identified prior to removal of duplicate records. After initial screening of titles and abstracts, 552 studies were selected for full-text assessment. Of these, 29 studies met the predefined inclusion criteria and were included in the narrative synthesis [22,33,34,35,36,37,38,39,40,41,42,43,44,45,46,47,48,49,50,51,52,53,54,55,56,57,58,59,60]. The flow chart of this selection procedure is presented in Figure 2.

### 3.2. Study Characteristics

Most studies were classified as retrospective cohort studies (*n* = 24), while a small number used cross-sectional (*n* = 2), prospective (*n* = 2), or qualitative designs (*n* = 1). Sample sizes ranged from 7 to 3220 participants (median 68), with 42.9% of studies including fewer than 50 participants. Studies were conducted in single-center, multi-center, and population-based settings and used medical records, registries, or patient- and clinician-reported data. Upper age limits differed across studies. Study characteristics are summarized in Table 2 and Supplementary Table S4.

### 3.3. Diagnostic Intervals

#### 3.3.1. Definitions and Measurement of Diagnostic Intervals

Studies varied considerably in the definitions and measurement of diagnostic intervals. Some studies reported only proportions [22,54,60], whereas others reported medians, ranges, or interquartile ranges. One study reported all summary statistics (means, medians, ranges, and interquartile ranges) [55].

Definitions of key diagnostic time points also varied across studies. For example, the date of diagnosis was defined as either the date of biopsy or the date of histopathological confirmation, while the definition of initial healthcare contact included presentations to general practitioners, emergency departments, or other healthcare providers. Several studies reported pathway-specific intervals, describing individual components of the diagnostic pathway. Definitions of diagnostic time points and reporting characteristics are summarized in Supplementary Tables S4 and S5.

#### 3.3.2. Reporting of the Five Predefined Diagnostic Intervals

**Figure 3:**
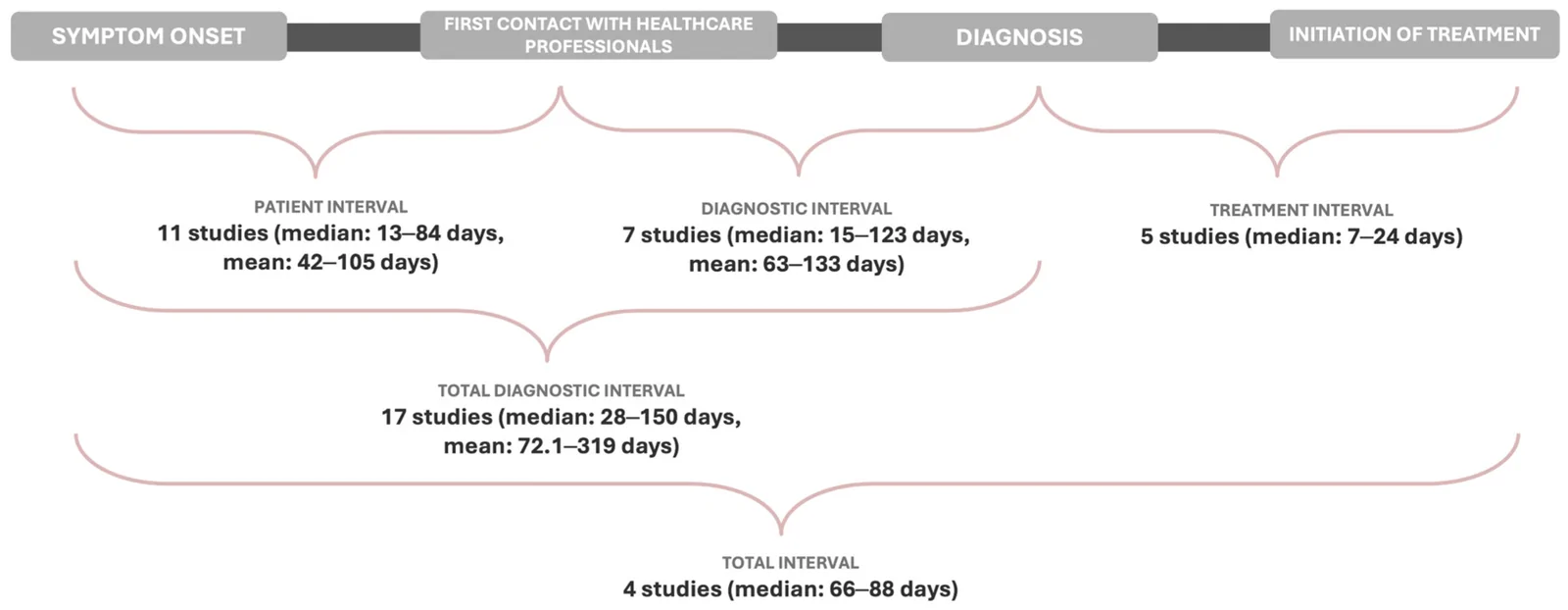
Frequency of Reported Diagnostic Intervals across Included Studies.

Twenty-five studies reported data on at least one of the five predefined diagnostic intervals. Eleven studies reported more than one of the intervals of interest [22,33,36,39,40,48,50,51,53,55,56]. Only one study reported all five predefined diagnostic intervals [39], limiting direct comparison of multiple predefined diagnostic intervals within individual studies. The frequency with which each predefined diagnostic interval was reported is shown in Figure 3.

#### 3.3.3. Duration of the Five Predefined Diagnostic Intervals

**Figure 4:**
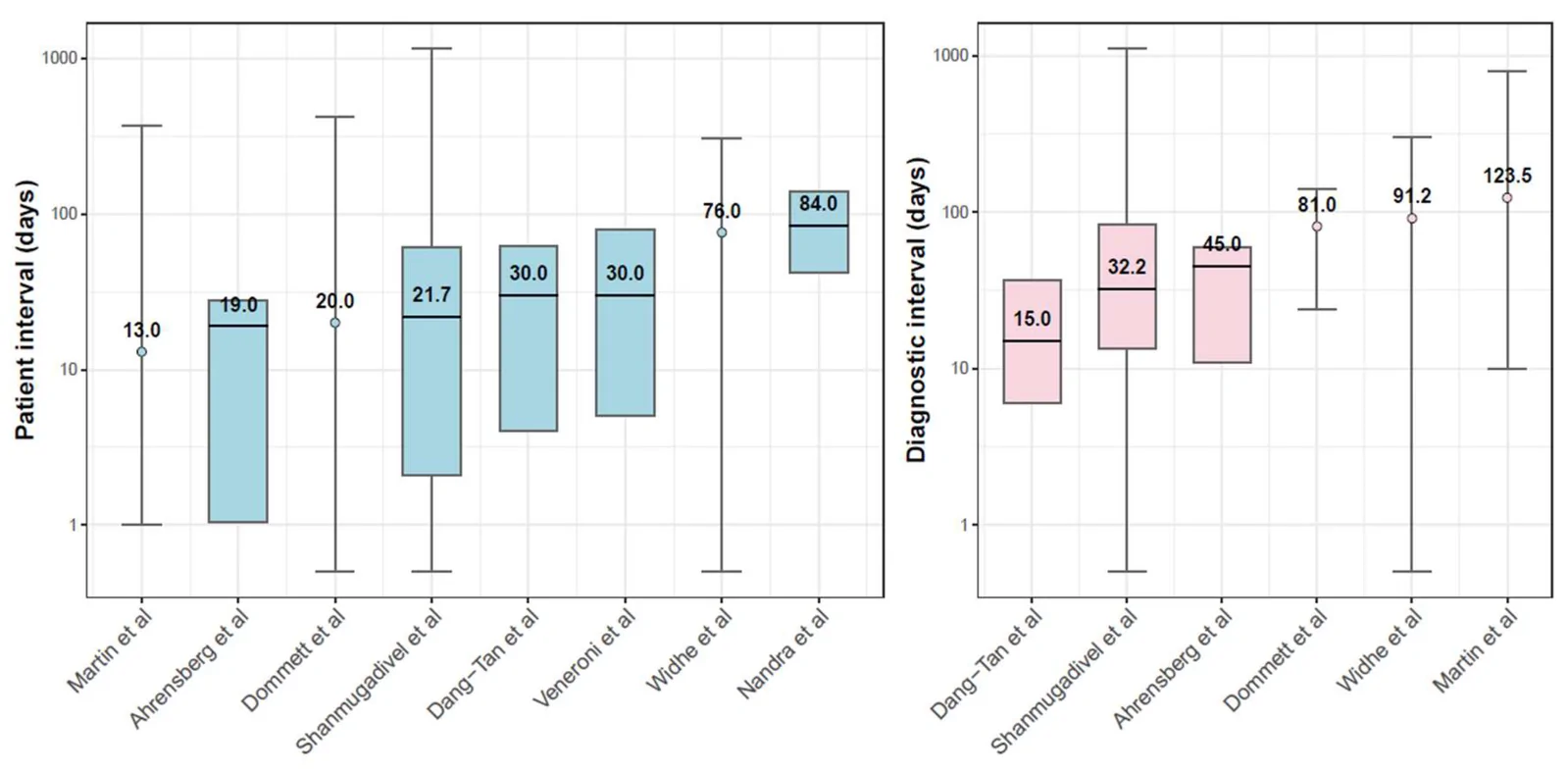

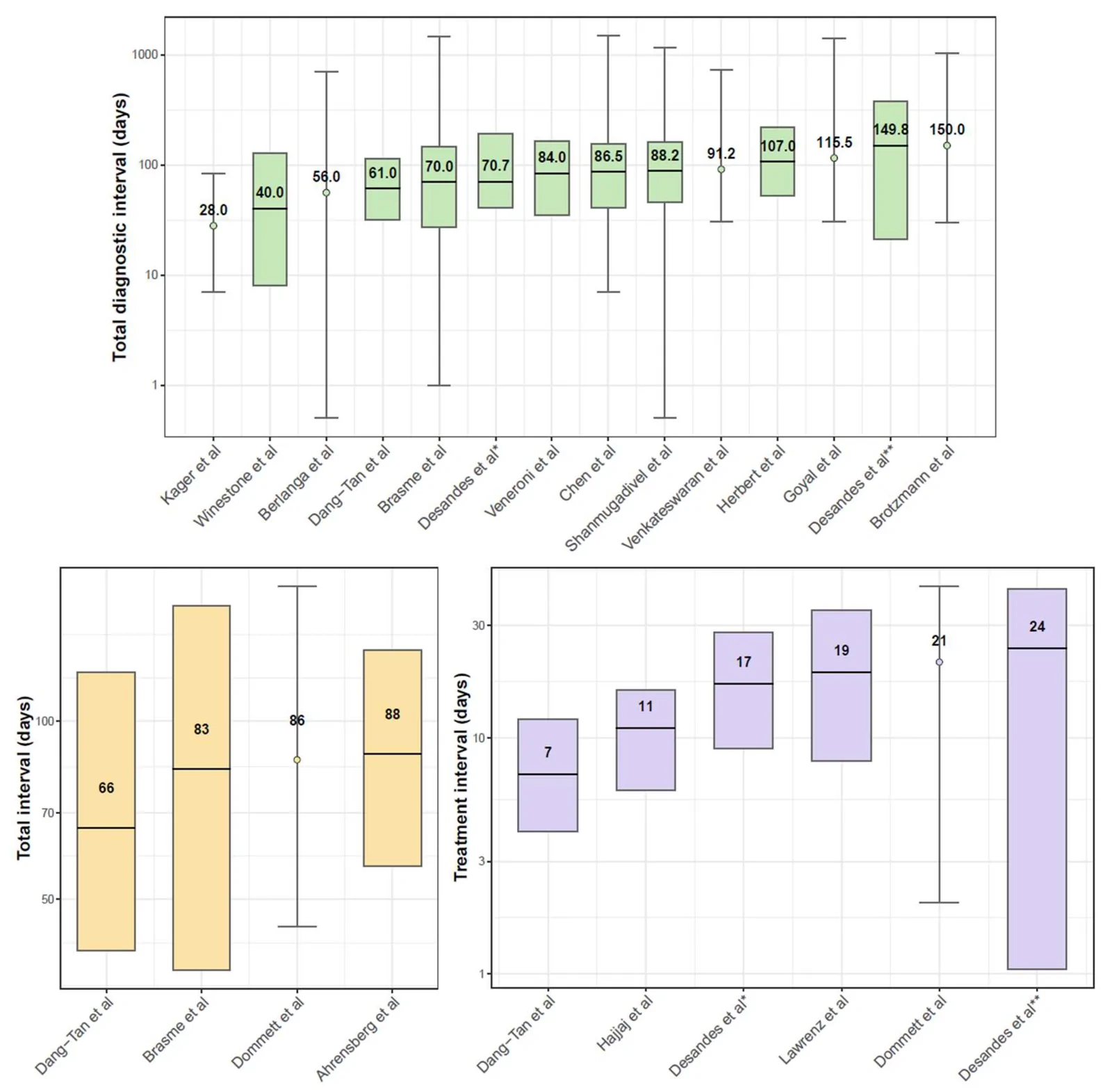
Median Values and Reported Distributions for the Five Diagnostic Intervals across Included Studies. Each boxplot represents the summary statistics reported in the original study. Numbers indicate the reported median interval for each study. Boxes represent the interquartile range and whiskers the reported range. The absence of a box or whiskers indicates that the corresponding summary measure was not reported in the study. * 15–19 years. ** 20–25 years [22,33,35,36,37,38,39,40,41,42,43,44,46,48,49,50,53,55,56].

Among studies reporting the five predefined diagnostic intervals, reported median durations varied substantially across all interval categories (Figure 4). Eleven studies reported the patient interval, with median durations ranging from 13 to 84 days [22,33,39,46,48,50,51,53,54,55,56]. Seven studies reported the diagnostic interval, with median durations ranging from 15 to 123 days [33,39,50,51,53,55,56]. The total diagnostic interval was the most frequently reported predefined diagnostic interval, assessed in 17 studies [22,34,^35^,^36^,^37^,^38^,^39^,^40^,41,^43^,45,^47^,^48^,^49^,51,^52^,^55^], with median durations ranging from 28 to 150 days. Five studies reported the treatment interval [39,40,42,44,53], with median durations ranging from 7 to 24 days, while four studies reported the total interval, with median durations ranging from 66 to 88 days [33,36,39,53]. The distributions of reported median durations across the five predefined diagnostic intervals are shown in Figure 4.

### 3.4. Pathway-Specific Intervals

Seven studies reported pathway-specific intervals defined using different time points along the diagnostic pathway (Table 3) [33,39,48,53,57,58,59]. Reported intervals ranged from broader measures spanning multiple stages of the diagnostic pathway to more specific intervals describing referral processes and specialist assessment.

Two studies reported primary care intervals, both of which were short (median of 0 and 1 days) [33,53]. In contrast, these studies reported longer intervals following referral, particularly during specialist assessment and diagnostic work-up. One study further reported a median interval of 44 days from initiation of a GP-led investigation to treatment initiation [33]. The only study judged to have low risk of bias [39] similarly indicated that a substantial proportion of the diagnostic pathway occurred after the initial healthcare contact.

### 3.5. Factors Associated with Diagnostic Interval Duration

#### 3.5.1. Patient-, Tumor-, and System-Related Factors

Studies examining patient-, tumor-, and system-related factors showed largely inconsistent associations with diagnostic interval duration. Older age within the CAYA population was the factor most consistently associated with longer diagnostic intervals [36,40,41,52,57]. Findings for sex were conflicting [36,50,51,52,57], while tumor site showed a more consistent pattern, with axial or central locations associated with longer diagnostic intervals [36,41,49,57].

No consistent associations were identified for metastasis status at diagnosis [36,41,57], tumor characteristics [41,51,57], treatment-related factors [36,57], or socioeconomic factors, including educational level and household income [52]. In contrast, some specific clinical features, such as the presence of a palpable mass, were associated with shorter diagnostic intervals in individual studies [36,50,51].

Overall, the most consistent evidence supported associations between older age, axial tumor location, and longer diagnostic intervals. These associations were observed across studies with low, moderate, and high risk of bias, whereas findings for other patient-, tumor-, and system-related factors remained inconsistent.

#### 3.5.2. Healthcare Entry Points and Sources of Prolonged Diagnostic Intervals

Six studies reported the distribution of entry points to the healthcare system [41,50,51,52,53,58]. Across the six studies, patients most commonly entered the healthcare system through primary care, with GPs accounting for the majority of initial healthcare contacts. Emergency department presentation was also reported, whereas presentation through other healthcare providers was less common. Reporting of healthcare entry points was incomplete, with many studies not specifying entry points or their distribution.

Reported findings suggested that prolonged diagnostic intervals may arise at multiple stages of the diagnostic pathway. Several studies described low suspicion of malignancy at initial presentation [38,50,51,53,54,56] and/or frequent initial misdiagnosis, with symptoms commonly attributed to benign musculoskeletal conditions [38,50,51,56]. Diagnostic imaging also appeared to influence diagnostic interval duration: radiography performed at the first medical visit was associated with shorter intervals in one low-risk study [51], whereas non-diagnostic or inconclusive initial imaging was associated with longer intervals in a moderate-risk study [58]. Several studies reported longer intervals after the initial healthcare contact, including during referral from primary to specialist care [22,48,53,54,56] and subsequent specialist assessment and diagnostic work-up [39,53,55].

Overall, the available evidence suggested that prolonged diagnostic intervals may accumulate across multiple stages of the diagnostic pathway rather than at a single point. This pattern was supported by the available low-risk-of-bias studies [39,51,55], although evidence for individual components of the diagnostic pathway remained limited.

### 3.6. Influence of Prolonged Diagnostic Intervals on Outcome

No consistent association between diagnostic interval duration and survival was observed across the four studies examining this association. Most studies [36,41,43] reported no significant relationship, although one study found improved survival among patients with very short intervals (<4 weeks), while no difference was observed when comparing longer intervals (>8 weeks) [57]. Overall, the available evidence did not support a consistent association between diagnostic interval duration and survival, although conclusions remain limited by the small number of studies.

### 3.7. Risk of Bias Across Studies

Three studies [36,39,55] were judged to have a low risk of bias, whereas the remaining studies were rated as having moderate or high risk of bias. High risk of bias was primarily driven by retrospective single-center study designs, potential recall bias, and selection bias. The complete assessment is presented in Supplementary Table S6.

## 4. Discussion

This systematic review synthesized evidence on diagnostic intervals in CAYA with primary bone sarcoma using five predefined diagnostic intervals supplemented by pathway-specific intervals. The predefined diagnostic intervals enabled standardized comparisons across studies despite substantial variation in interval definitions and reporting, while the pathway-specific intervals provided additional insight into where prolonged intervals occurred within the diagnostic pathway. Together, these approaches enabled standardized comparison while providing additional insight into diagnostic pathways.

Considerable variability was observed across all five predefined diagnostic intervals. Although this likely reflects methodological differences between studies, differences in healthcare organization and diagnostic pathways probably also contributed.

The patient interval ranged from a median of 13 to 84 days, indicating substantial variation in the time from symptom onset to initial healthcare contact. This may partly reflect differences in symptom appraisal and help-seeking behavior within the CAYA population. A recent qualitative systematic review supports this interpretation, reporting that patients with sarcoma frequently normalized or attributed early symptoms to benign causes, thereby delaying healthcare seeking [61].

Across studies reporting healthcare entry points, primary care was the most common initial healthcare contact. Similarly, Gerrand et al. [62] reported that most CAYA with bone sarcoma were referred through general practitioners, whereas younger children (<10 years) more frequently presented as emergencies, suggesting that diagnostic pathways may differ by age.

Although evidence was limited, the available pathway-specific data suggested that primary care intervals were generally short, whereas longer intervals appeared to occur after referral during specialist assessment and diagnostic work-up. Several studies also described low suspicion of malignancy at initial presentation, frequent initial misdiagnosis, and referral to non-cancer pathways, indicating that prolonged diagnostic intervals may reflect cumulative contributions from multiple stages of the diagnostic pathway. While this overall pattern was also observed in the available low-risk-of-bias studies, evidence for the relative contribution of individual pathway components remains limited.

The pathway analysis by Dommett et al. [53] provides further insight into this pattern. Despite short primary care intervals, patients were frequently referred promptly to secondary care without suspicion of malignancy, resulting in sequential investigations and repeated multidisciplinary discussions before diagnosis. Martin et al. [56] similarly highlighted low suspicion of malignancy and frequent initial misdiagnosis, supporting this overall pattern.

A similar pattern has also been described in an adult Swiss sarcoma population, where the secondary care interval constituted a substantial part of the overall diagnostic pathway [63].

In contrast to the pathway-specific findings, associations between diagnostic interval duration and patient- and tumor-related factors were generally inconsistent. Older age within the CAYA population was the only patient-related factor consistently associated with longer diagnostic intervals across studies. Interpretation of this finding was, however, complicated by substantial variation in age group definitions. Given the broad age range encompassed by the CAYA population, more consistent and clinically meaningful age stratification is needed to better characterize age-specific diagnostic pathways. This is particularly important because older age is also an established adverse prognostic factor in bone sarcoma [7,8].

Findings regarding sex were inconsistent, ranging from longer diagnostic intervals in females to no association or opposite trends. However, recent population-based evidence suggests that help-seeking patterns differ between males and females [64], indicating that sex-specific differences in diagnostic pathways warrant further investigation.

No consistent association between diagnostic interval duration and survival was identified. While three studies reported no significant relationship, one study found improved survival among patients with very short intervals (<4 weeks), with no corresponding difference observed for longer intervals. These findings are consistent with the previous literature suggesting that the relationship between diagnostic interval duration and survival is complex and difficult to interpret [21,65]. The small number of available studies, together with methodological heterogeneity and variation in reported outcomes, precludes firm conclusions regarding the prognostic significance of diagnostic interval duration.

Although the available evidence remains limited and heterogeneous, this review suggests that prolonged diagnostic intervals may arise across multiple stages of the diagnostic pathway rather than solely before the initial healthcare contact. These findings highlight potential areas for further evaluation, including follow-up of persistent or unexplained musculoskeletal symptoms, timely diagnostic imaging, and specialist assessment. However, the effectiveness of these approaches in reducing diagnostic intervals has not been established. Furthermore, the observed associations between palpable mass, early imaging, and shorter diagnostic intervals identify potentially important hypotheses that warrant further investigation. Finally, the consistent association between older age and longer diagnostic intervals suggests that age-specific diagnostic pathways within the CAYA population deserve further attention in both clinical practice and future research.

### Strengths and Limitations

This review presents a comprehensive synthesis of diagnostic intervals and associated factors in CAYA with primary bone sarcoma across multiple healthcare settings. To our knowledge, this is the first systematic review to focus specifically on this patient population. Previous evidence has only been synthesized as part of broader reviews of pediatric cancer or sarcoma.

Several limitations should be considered when interpreting the findings. Most included studies were retrospective and relied on medical chart reviews, questionnaires, or interviews to determine the symptom onset. These methods are vulnerable to recall, selection, and survivor bias, while medical chart-based studies depend on the completeness of clinical documentation. As a result, symptom onset is an imprecise reference point for both the patient interval and the total diagnostic interval, which reduces the accuracy and comparability of estimates across studies.

Further methodological heterogeneity arose from variation in the definition of key diagnostic time points, including symptom onset and date of diagnosis (e.g., date of biopsy [34,36,42,43] vs. histopathological confirmation [37,44,56]), as well as inconsistent inclusion criteria (e.g., only inclusion of patients with symptoms in the 6 months prior to diagnosis [52]). In addition, many studies were small, more than one-third were single-center studies, and only three studies were judged to have low risk of bias, limiting the certainty of the available evidence. Several included studies reported broader cohorts of primary bone sarcomas or malignant bone tumors without providing histology-specific patient numbers or interval data, precluding meaningful comparisons between osteosarcoma and Ewing sarcoma.

Finally, only studies from HICs were included. Consequently, the findings may not be applicable to LMICs, where healthcare infrastructure, diagnostic pathways, and access to specialist care may differ substantially. The reported diagnostic intervals and contributing factors should therefore be interpreted within the context of HIC healthcare systems.

Future studies should prioritize prospective study designs, standardized definitions of diagnostic time points, adherence to established interval-reporting frameworks (such as the REST guidance [31] and the Aarhus Statement [25]), and transparent reporting of interval start and end points. More consistent reporting of diagnostic intervals and diagnostic pathways will facilitate comparison across studies and strengthen the evidence base for understanding diagnostic timeliness in CAYA with primary bone sarcoma.

## 5. Conclusions

This systematic review synthesized evidence on diagnostic intervals in CAYA with primary bone sarcoma using five predefined diagnostic intervals supplemented by pathway-specific intervals. Reported diagnostic interval durations varied substantially across studies, reflecting both methodological heterogeneity and differences in diagnostic pathways and healthcare systems. Pathway-specific intervals provided additional insight into different components of the diagnostic pathway, particularly after initial healthcare contact, although evidence remained limited. Older age within the CAYA population and axial tumor location were the factors most consistently associated with longer diagnostic intervals. Future prospective studies using standardized interval definitions and reporting are needed to improve understanding and strengthen the evidence base.

## Figures and Tables

**Table 1:** Definition of Diagnostic Intervals Based on the Anderson Model [24].

Interval	Definition
Patient interval	Period from the first symptom until the initial contact with healthcare professionals ^1^
Diagnostic interval	Period from initial contact with healthcare professionals ^1^ until diagnosis
Total diagnostic interval	Period from the first symptom until diagnosis
Treatment interval	Period from diagnosis until the initiation of treatment
Total interval	Period from the first symptom until the initiation of treatment

^1^ Initial healthcare presentation denotes the earliest clinical encounter at which investigation or referral could have been initiated.

**Table 2:** Characteristics of the Included Studies, including study design, data sources, and histological subtypes.

Characteristics	*n*	%
Publication year		
2000–2009	8	27.6
2010–2019	13	44.8
2020–2025	8	27.6
Setting		
Anglo-American countries	17	58.6
Central European countries	4	13.8
Southern European countries	5	17.2
Nordic countries	3	10.3
Type of study population		
Multi-center	10	34.5
Single-center	13	44.8
Population-based	6	20.7
Type of study		
Retrospective cohort study	24	82.8
Cross-sectional observational study	2	6.9
Qualitative study	1	3.4
Prospective observational/cohort study	2	6.9
Type of data source		
Medical charts	20	69.0
National/regional register/database	10	34.5
Patient/parent/GP interviews/questionnaires/forms	8	27.6
Institutional/hospital database	4	13.8
Clinical trial data	1	3.4
Type of tumors		
Any bone tumors/sarcomas	19	65.5
Osteosarcomas only	6	20.7
Ewing sarcomas only	4	13.8

Values are presented as number of studies (*n*) and percentage of included studies (%). Categories under “Types of data source” are not mutually exclusive; therefore, studies may be represented in more than one category. Abbreviation: GP, general practitioner.

**Table 3:** Studies Reporting Additional Pathway-Specific Intervals.

Ref.	Interval(s) Reported
[33]	Median GP interval (first symptom presentation to the GP to initiation of an investigation to determine the presence of a potential cancer): 0 days (IQR: 0–17 days)
	Median system interval (start of the GP-initiated investigation until the start of treatment): 44 days (IQR: 26–73 days)
[39]	Median referral interval (first medical contact to assessment by oncologist): 12 days (IQR: 2–35)
	Median oncologist interval (assessment by oncologist to cancer diagnosis): 4 days (IQR: 1–6 days) Median healthcare system interval (first medical contact to start of treatment): 24 days (IQR: 13–48 days)
[53]	Median doctor interval (first presentation/clinical appearance to first investigation): 1 day (range: 0–80 days)
	Median primary care interval (first presentation/clinical appearance to first referral): 1 day (range: 0–63 days) Median referral interval (first referral to first specialist visit): 34 days (range: 0–61 days) Median secondary care interval (from first referral to start of treatment): 79 days (range: 24–168 days) Median specialist care interval (first specialist visit to start of treatment): 30 days (range: 24–109 days) Median system interval (first investigation to start of treatment): 80 days (range: 6–169 days)
[59]	Median interval to specialist assessment (onset of symptoms to first review at a specialist orthopedic center): 35 days (IQR: 17–88)
[58]	Mean interval to diagnosis (first presentation to a specialist orthopedic hospital to pathology-confirmed diagnosis): 19.5 days (SD: 65; range: 0–493 days)
[57]	Median interval to specialist assessment (onset of symptoms to first review at a specialist orthopedic center): 56 days * (IQR: 28–84 days *)
[48]	Median referral interval (first doctor’s visit to assessment by an oncologist): 24 days (IQR: 9–85 days)

* Converted from weeks to days by multiplying with 7.

## Data Availability

No new data were created or analyzed in this study. Data sharing is not applicable to this article.

## Supplementary Materials

The following supporting information can be downloaded at: https://www.mdpi.com/article/10.3390/cancers18162590/s1, Table S1: Search Strings MEDLINE and Embase; Table S2: Selection Criteria; Table S3: Risk of Bias Assessment Template; Table S4: Study Characteristics; Table S5: Reported Intervals Among Selected Studies; Table S6: Risk of Bias Assessment across Included Studies.

## Abbreviations

The following abbreviations are used in this manuscript:

CAYA	Children, adolescents, and young adults
GP	General practitioner
LMICs	Low- and middle-income countries
HICs	High-income countries
