# Supplementary material for: Diagnostic Intervals in Children, Adolescents and Young Adults with Primary Bone Sarcoma: A Systematic Review
Source: Cancers (Basel). 2026 Aug 12;18(16):2590. doi: 10.3390/cancers18162590 (PMC13510990; doi:10.3390/cancers18162590)
Supplement: Supplementary file 1 [file cancers-18-02590-s001.zip › Supplementary Table S1.pdf]

Supplementary Table S1. Search Strings in MEDLINE and Embase.

**Search string MEDLINE**	
Search name	*Search query*
Block 1: Intervals / diagnostic delay	MeSH terms and keywords
1	(exp delayed diagnosis/ OR exp early diagnosis/ OR exp Time-to-Treatment/ OR ((delay* OR late* OR early OR earlie* OR barrier* OR interval* OR time* OR route*) adj4 (diagnos* OR referal* OR referral* OR treatment* OR therap*)).tw,kf.)
Block 2: Bone sarcoma	MeSH terms and keywords
2	(exp osteosarcoma/ OR exp bone neoplasms/ OR ((“bone” OR “osse?us” OR osteogen* OR skelet*) adj4 (neoplasm* OR cancer* OR tumo?r* OR malignan* OR sarcoma*)).tw,kf. OR (ewing* OR osteosarcoma* OR chondrosarcoma*).tw,kf.)
Block 3: Only humans	MeSH terms
3	exp animal/
4	exp human/
5	exp animal/ NOT exp human/
Block 4: Only primary bone tumors	MeSH terms
6	exp neoplasm metastasis/
7	(exp leukemia/ OR exp lymphatic vessel tumors/ OR exp lymphoma/ OR exp “neoplasms, glandular and epithelial"/ OR exp neoplasms, gonadal tissue/ OR exp neoplasms, plasma cell/ OR exp "nevi and melanomas"/ OR exp anal gland neoplasms/ OR exp breast neoplasms/ OR exp digestive system neoplasms/ OR exp endocrine gland neoplasms/ OR exp hematologic neoplasms/ OR exp mammary neoplasms, animal/ OR exp skin neoplasms/ OR exp soft tissue neoplasms/ OR exp splenic neoplasms/ OR exp urogenital neoplasms/)
8	exp neoplasm metastasis/ AND (exp leukemia/ OR exp lymphatic vessel tumors/ OR exp lymphoma/ OR exp “neoplasms, glandular and epithelial"/ OR exp neoplasms, gonadal tissue/ OR exp neoplasms, plasma cell/ OR exp "nevi and melanomas"/ OR exp anal gland neoplasms/ OR exp breast neoplasms/ OR exp digestive system neoplasms/ OR exp endocrine gland neoplasms/ OR exp hematologic neoplasms/ OR exp mammary neoplasms, animal/ OR exp skin neoplasms/ OR exp soft tissue neoplasms/ OR exp splenic neoplasms/ OR exp urogenital neoplasms/)
Final search:	
1 AND 2 NOT 5 NOT 8 AND filter(from 2002/1/1-2025/2/11)	(exp delayed diagnosis/ OR exp early diagnosis/ OR Time-to-Treatment/ OR ((delay* OR late* OR early OR earlie* OR barrier* OR interval* OR time* OR route*) adj4 (diagnos* OR referal* OR referral* OR treatment* OR therap*)).tw,kf.) AND (exp osteosarcoma/ OR exp bone neoplasms/ OR ((“bone” OR “osse?us” OR osteogen* OR skelet*) adj4 (neoplasm* OR cancer* OR tumo?r* OR malignan* OR sarcoma*)).tw,kf. OR (ewing* OR osteosarcoma* OR chondrosarcoma*).tw,kf.) NOT (exp animal/ NOT exp human/) NOT (exp neoplasm metastasis/ AND (exp leukemia/ OR exp lymphatic vessel tumors/ OR exp lymphoma/ OR exp “neoplasms, glandular and epithelial"/ OR exp neoplasms, gonadal tissue/ OR exp neoplasms, plasma cell/ OR exp "nevi and melanomas"/ OR exp anal gland neoplasms/ OR exp breast neoplasms/ OR exp digestive system neoplasms/ OR

	exp endocrine gland neoplasms/ OR exp hematologic neoplasms/ OR exp mammary neoplasms, animal/ OR exp skin neoplasms/ OR exp soft tissue neoplasms/ OR exp splenic neoplasms/ OR exp urogenital neoplasms/)) AND (2002/1/1:2025/1/1[pdat])

**Search string Embase**	
Search name	*Search query*
Block 1: Intervals / diagnostic delay	MeSH terms and keywords
1	exp diagnosis time/ OR exp diagnosis to treatment interval/ OR exp delayed diagnosis/ OR exp early diagnosis/ OR exp therapy delay/ OR exp time to treatment OR ((delay* OR late* OR early OR earlie* OR barrier* OR interval* OR time* OR route*) adj4 (diagnos* OR referal* OR referral* OR treatment* OR therap*)).tw,kf.
Block 2: Bone sarcoma	MeSH terms and keywords
2	exp bone cancer/ OR exp bone tumor/ OR exp bone sarcoma/ OR exp osteosarcoma/ OR exp Ewing sarcoma/ OR exp chondrosarcoma/ OR ((bone OR osse?us OR osteogen* OR skelet*) adj4 (neoplasm* OR cancer* OR tumo?r* OR malignan* OR sarcoma*)).tw,kf. OR (Ewing* OR osteosarcoma* OR chondrosarcoma*).tw,kf.
Block 3: Only humans	MeSH terms
3	exp animal/
4	exp human/
5	exp animal/ NOT exp human/
Block 4: Only primary bone tumors	MeSH terms
6	exp metastasis/
7	exp hematologic malignancy/ OR exp hematopoietic system malignancy/ OR exp lymphatic system malignancy/ OR exp gonad tumor/ OR exp “nevi and melanomas”/ OR exp “glandular and epithelial neoplasms”/ OR exp breast cancer/ OR exp anus tumor/ OR exp digestive system tumor/ OR exp endocrine tumor/ OR exp skin tumor/ OR exp spleen tumor/ OR exp urogenital tract tumor/
8	exp metastasis/ AND (exp hematologic malignancy/ OR exp hematopoietic system malignancy/ OR exp lymphatic system malignancy/ OR exp gonad tumor/ OR exp “nevi and melanomas”/ OR exp “glandular and epithelial neoplasms”/ OR exp breast cancer/ OR exp anus tumor/ OR exp digestive system tumor/ OR exp endocrine tumor/ OR exp skin tumor/ OR exp spleen tumor/ OR exp urogenital tract tumor/)
Final search:	
1 AND 2 NOT 5 NOT 8 AND filter (publication year=”2000-2025”)	(exp diagnosis time/ OR exp diagnosis to treatment interval/ OR exp delayed diagnosis/ OR exp early diagnosis/ OR exp therapy delay/ OR exp time to treatment OR ((delay* OR late* OR early OR earlie* OR barrier* OR interval* OR time* OR route*) adj4 (diagnos* OR referal* OR referral* OR treatment* OR therap*)).tw,kf.) AND (exp bone cancer/ OR exp bone tumor/ OR exp bone sarcoma/ OR exp osteosarcoma/ OR exp Ewing sarcoma/ OR exp chondrosarcoma/ OR ((bone OR osse?us OR osteogen* OR skelet*) adj4

	(neoplasm* OR cancer* OR tumo?r* OR malignan* OR sarcoma*)).tw,kf. OR (Ewing* OR osteosarcoma* OR chondrosarcoma*).tw,kf.) NOT ((exp animal/) NOT (exp human/)) NOT ((exp metastasis/) AND (exp hematologic malignancy/ OR exp hematopoietic system malignancy/ OR exp lymphatic system malignancy/ OR exp gonad tumor/ OR exp "nevi and melanomas"/ OR exp "glandular and epithelial neoplasms"/ OR exp breast cancer/ OR exp anus tumor/ OR exp digestive system tumor/ OR exp endocrine tumor/ OR exp skin tumor/ OR exp spleen tumor/ OR exp urogenital tract tumor/)) AND (2002:py OR 2003:py OR 2004:py OR 2005:py OR 2006:py OR 2007:py OR 2008:py OR 2009:py OR 2010:py OR 2011:py OR 2012:py OR 2013:py OR 2014:py OR 2015:py OR 2016:py OR 2017:py OR 2018:py OR 2019:py OR 2020:py OR 2021:py OR 2022:py OR 2023:py OR 2024:py OR 2025:py OR)

**Search string Cochrane Central Register of Controlled Trials (CENTRAL)**	
Search name	*Search query*
Block 1: Intervals / diagnostic delay	MeSH terms and keywords
1	(MeSH descriptor: [Delayed Diagnosis] explode all trees) OR (MeSH descriptor: [Early Diagnosis] explode all trees) OR ("diagnostic delay" OR "diagnostic interval" OR "time to diagnosis")
Block 2: Bone sarcoma	MeSH terms and keywords
2	(MeSH descriptor: [Osteosarcoma] explode all trees) OR (MeSH descriptor: [Bone Neoplasms] explode all trees) OR ((bone OR osseous OR osteogen* OR skeletal) AND (neoplasm* OR cancer* OR tumor* OR tumour* OR malignan* OR sarcoma*)) OR (ewing* OR osteosarcoma* OR chondrosarcoma*)
Final search:	Publication date filter: 1 January 2002 to 1 February 2025
1 AND 2	((MeSH descriptor: [Delayed Diagnosis] explode all trees) OR (MeSH descriptor: [Early Diagnosis] explode all trees) OR ("diagnostic delay" OR "diagnostic interval" OR "time to diagnosis")) AND ((MeSH descriptor: [Osteosarcoma] explode all trees) OR (MeSH descriptor: [Bone Neoplasms] explode all trees) OR ((bone OR osseous OR osteogen* OR skeletal) AND (neoplasm* OR cancer* OR tumor* OR tumour* OR malignan* OR sarcoma*)) OR (ewing* OR osteosarcoma* OR chondrosarcoma*))

**Search string Web of Science**	
Search name	*Search query*
Block 1: Intervals / diagnostic delay	
1	TS=("diagnostic delay" OR "diagnostic interval" OR "time to diagnosis" OR "delayed diagnosis")
Block 2: Bone sarcoma	

2	TS=(osteosarcoma* OR "ewing sarcoma*" OR "ewing tumor*" OR chondrosarcoma* OR "primary bone sarcoma*" OR ("bone" NEAR/3 sarcoma*))
Final search:	Publication year filter: 2002 to 2025
1 AND 2	TS=(("diagnostic delay" OR "diagnostic interval" OR "time to diagnosis" OR "delayed diagnosis") AND (osteosarcoma* OR "ewing sarcoma*" OR "ewing tumor*" OR chondrosarcoma* OR "primary bone sarcoma*" OR ("bone" NEAR/3 sarcoma*)))

**Search string Scopus (Elsevier)**	
Search name	*Search query*
Block 1: Intervals / diagnostic delay	
1	TITLE-ABS-KEY("diagnostic delay" OR "diagnostic interval" OR "time to diagnosis" OR "delayed diagnosis")
Block 2: Bone sarcoma	
2	TITLE-ABS-KEY(osteosarcoma* OR "ewing sarcoma*" OR "ewing tumor*" OR chondrosarcoma* OR "primary bone sarcoma*" OR "bone sarcoma")
Final search:	Publication year filter: 2002 to 2025
1 AND 2	TITLE-ABS-KEY(("diagnostic delay" OR "diagnostic interval" OR "time to diagnosis" OR "delayed diagnosis") AND (osteosarcoma* OR "ewing sarcoma*" OR "ewing tumor*" OR chondrosarcoma* OR "primary bone sarcoma*" OR "bone sarcoma"))
