# Supplementary material for: Diagnostic Intervals in Children, Adolescents and Young Adults with Primary Bone Sarcoma: A Systematic Review
Source: Cancers (Basel). 2026 Aug 12;18(16):2590. doi: 10.3390/cancers18162590 (PMC13510990; doi:10.3390/cancers18162590)
Supplement: Supplementary file 1 [file cancers-18-02590-s001.zip › Supplementary Table S2.pdf]

Supplementary Table S2. Selection Criteria.

Inclusion Criteria	Exclusion Criteria
**Population:** Minimum of five study participants diagnosed with a primary bone sarcoma, aged 0–39 years	**Population:** Populations with known sarcoma predisposition syndromes, secondary malignancies, sub-populations less than five
**Countries included:** High-income countries, as defined by the World Bank's 2024–2025 income classification	**Type of publication:** Conference abstracts, case studies, literature review, review, meta-analysis, systematic review, editorials, commentaries, letters
**Primary outcomes:** Patient interval, diagnostic interval, total diagnostic interval, treatment interval, total interval, and/or additional pathway-specific intervals	**Area of Study:** Upper–middle-, lower–middle-, and low-income countries, as defined by the World Bank's 2024–2025 income classification
**Publication dates:** 01/01/2000 – 11/02/2025 *	**Study focus:** Diagnosis via genetic testing only
**Type of publication:** Original research articles (presenting primary data)	
**Language:** English	

* One additional study published after the predefined end date (May 2025) was identified through forward citation searching of included articles and was therefore included.
