# Supplementary material for: Diagnostic Intervals in Children, Adolescents and Young Adults with Primary Bone Sarcoma: A Systematic Review
Source: Cancers (Basel). 2026 Aug 12;18(16):2590. doi: 10.3390/cancers18162590 (PMC13510990; doi:10.3390/cancers18162590)
Supplement: Supplementary file 1 [file cancers-18-02590-s001.zip › Supplementary Table S3.pdf]

Supplementary Table S3. Risk of Bias Assessment Template.

Study ID	Domain	Assessment focus	Risk of Bias (High / Moderate / Low)	Comment
	1. Study population representativeness and reporting	Evaluate how the study population was selected, and whether its characteristics are clearly described and representative of bone sarcoma patients aged 0–39.		
	2. Definition and measurement of diagnostic intervals	Evaluate the clarity, consistency, and reliability of how diagnostic time points and intervals are defined and measured, including the credibility of data sources from which interval estimates are derived.		
	3. Data completeness: Reporting of diagnostic intervals	Evaluate whether all relevant diagnostic intervals are reported with sufficient detail.		
	4. Reporting transparency and handling of time data	Evaluate how clearly and completely the study reports interval data, including how missing or extreme values are handled.		
	5. Bias and confounding considerations	Evaluate awareness of and control for potential biases.		
