# Supplementary material for: Diagnostic Intervals in Children, Adolescents and Young Adults with Primary Bone Sarcoma: A Systematic Review
Source: Cancers (Basel). 2026 Aug 12;18(16):2590. doi: 10.3390/cancers18162590 (PMC13510990; doi:10.3390/cancers18162590)
Supplement: Supplementary file 1 [file cancers-18-02590-s001.zip › Supplementary Table S4.pdf]

Supplementary Table S4. Study Characteristics.

Author [ref]	Year	Study Design & Country	Data Collection Period	Study Population	Data Source	Histological Subtype(s) Included	Age Groups (years)	Sample Size	Factors Influencing Diagnostic Intervals and Delay	Healthcare Contacts Involved and Initial Point of Entry	Risk of Bias
Ahrensberg et al. [33]	2012	Retrospective cohort study<br>Denmark	2007-2010	Population-based	National registry, parent+GP questionnaires	All bone tumors	<15	315	Not reported.	GP involvement in the diagnostic pathway was reported in 19 out of 22 patients.	Moderate
Bacci et al. [34]	2000	Retrospective cohort study<br>Italy	1983-1999	Single-center	Medical charts	Osteosarcomas of the extremities	<20	607	Not reported.	Not reported.	High
Berlanga et al. [35]	2015	Retrospective cohort study<br>Spain	1985-2011	Single-center	Institutional database, medical charts	Osteosarcomas	≤22	85	Not reported.	Not reported.	Moderate to high
Brasme et al. [36]	2014	Retrospective cohort study	1988-2000	Multicenter	Trial data from two multicenter	Ewing sarcomas	<21	436	Not reported.	Not reported.	Low

		France			clinical trials						
Brot-zmann et al. [37]	2013	Retro-spective cohort study<br><br>Switzer-land	1969-2008	Multi-center	The Bone Tumor Refer-ence Center (BTCR) database, GP ques-tionnaire	Osteosar-comas, Ewing sarcomas and chondro-sarcomas of the foot	All ages	13	Not reported.	Not reported.	High
Chen et al. [38]	2017	Retro-spective cohort study<br><br>USA	2004-2012	Single-center	Medical charts	All bone tumors	<25	30	High rate of initial misdi-agnosis among bone tu-mor patients (53 %), with musculoskeletal pain fre-quently attributed to trauma or inflammation.	Not reported.	High
Dang-Tan et al. [39]	2008	Retro-spective cohort study<br><br>Canada	1995-2000	Popula-tion-based	National registry, medical charts	All bone tumors	<20	145	Total diagnostic delay likely driven by healthcare provider fac-tors, with long referral in-tervals and short patient delay.	Not reported.	Low
De-sandes et al. [40]	2018	Retro-spective cohort study	2012-2013	Popula-tion-based	Regional registry, medical charts	All bone tumors	15-19 and 20-24	48	Not reported.	Reported for overall co-hort only; not specified for bone sarcoma pa-tients.	Moder-ate

		France									
Dommett et al. [53]	2019	Retrospective cohort study †<br><br>England	2014-2016	Multicenter	Medical charts	All bone tumors	15-24	7	Cancer not suspected at first presentation in any patients (7/7), contributing to delayed diagnostic evaluation.<br><br>Initial referral to non-cancer pathways (e.g., musculoskeletal services). Delays primarily occurred after referral, with prolonged specialist care intervals including sequential investigations and delayed specialist review despite short primary care intervals.<br><br>No pathways classified as best practice by expert review, indicating multiple missed opportunities for earlier diagnosis.	Initial point of entry: Primary care (6/7) and Accident & Emergency department (1/7).	High
Ferrari et al. [60]	2015	Prospective cohort study<br><br>Italy	2007-2011	Single-center	Patient-/guardian-reported data collected at admission (ad hoc form)	Ewing sarcomas and osteosarcomas	Pediatric population**	45	Not reported.	Not reported.	Moderate

Goyal et al. [41]	2004	Retro-spective cohort study<br><br>England	1990-2002	Multi-center	Medical charts	Osteosar-comas and Ewing sarcomas	≤22	103	Initial presentation to GP associated with longer delay compared to A&E (4.3 vs 2.8 months).	Initial presentation to GP (50%), Accident & Emer-gency department (36%), consultant (5%), other professionals including rheumatologist and phys-iotherapist; 7% of pa-tients not specified.	Moder-ate
Hajjaj et al. [42]	2024	Retro-spective cohort study<br><br>Canada	2000-2018	Multi-center	Institu-tional clinical database, medical charts	Ewing sarcomas	≤18	41	Not reported.	Not reported.	Moder-ate
Herbert et al. [22]	2018	Cross-sectional observa-tional study<br><br>England	2012-2015	Multi-center	Patient inter-views, national cancer registry, medical charts	All ma-lignant bone tu-mors	12-24	68	Patients with bone tu-mors were more likely to have multiple pre-referral GP consultations (>3) compared to other cancer types, suggesting delayed escalation to specialist re-ferral.	First healthcare contact defined as presentation to GP or Accident & Emer-gency; no data on distri-bution reported.	Moder-ate–high
Kager et al. [43]	2010	Retro-spective cohort study<br><br>Ger-many, Austria and	1977-2005	Multi-center	National multi-center registry, medical charts	Osteosar-comas	<5	24	Not reported.	Not reported.	High

		Switzer-land									
Kell et al. [59]	2024	Retro-spective cohort study<br><br>USA	2006-2022	Single-center	Medical charts	Osteosar-comas	≤18	109	Not reported.	Not reported.	Moder-ate–high
Kha et al. [58]	2023	Retro-spective cohort study<br><br>USA	2004-2020	Single-center	Medical charts	Osteosar-comas and Ewing sarcomas	≤18	71	Negative initial radio-graphs (20%) were associ-ated with significantly prolonged diagnostic in-tervals compared to posi-tive findings, with delays persisting until further imaging (e.g., MRI) iden-tified malignancy.	Within the study hospi-tal: outpatient pediatric services (81%) and Emer-gency Department (18%).	Moder-ate
Lawrenz et al. [44]	2018	Retro-spective cohort study<br><br>USA	2004-2013	Multi-center	National database	All bone sarcomas	18-30	3,220	Not reported.	Not reported.	Low–moder-ate
Martin et al. [45]	2007	Retro-spective cohort study<br><br>USA	2001-2003	Single-center	Hospital registry, medical charts	All bone sarcomas	15-29	29	Not reported.	Not reported.	Moder-ate

Martin et al. [56]	2023	Qualitative study<br><br>England and Scotland	2017	Multi-center	Patient interviews	All bone sarcomas	25-39	10	Misdiagnosis and initial management (e.g., physiotherapy, presumed benign conditions) contributed to delayed diagnosis; missed imaging and incidental detection were also reported.	Not reported.	High
Nandra et al. [46]	2015	Retrospective cohort study<br><br>England	1985-2010	Single-center	Institutional database	All bone sarcomas	<22	1,367	Not reported.	Not reported.	High
Ruggieri et al. [47]	2009	Retrospective cohort study<br><br>Italy	1959-1999	Single-center	Medical charts	Osteosarcomas of the hip	<15	27	Not reported.	Not reported.	High
Shanmugadivel et al. [55]	2025	Prospective observational study<br><br>United Kingdom	2020-2023	Multi-center	Parent questionnaires, medical charts	All bone tumors	≤18	124	~34% of patients had >4 healthcare professional visits prior to diagnosis.	Not reported.	Low

Veneroni et al. [48]	2013	Retro-spective cohort study<br><br>Italy	2007-2011	Single-center	Medical charts, patient or parent question-naires	All bone sarcomas	Pediatric popula-tion**	65	Not reported.	First healthcare contact defined as first contact with any physician (e.g., GP/pediatrician, emer-gency physician, non-on-cology specialist, radiolo-gist); no data on distribu-tion across entry points.	Moder-ate
Venka-teswa-ran et al. [49]	2001	Retro-spective cohort study<br><br>USA	1962-1999	Single-center	Medical charts	Ewing sarcoma of the vertebrae	<22	33	Not reported.	Not reported.	High
Widhe et al. †† [50]	2007	Retro-spective cohort study<br><br>Sweden	1981-2000	Popula-tion-based	National register, medical charts	Ewing sarcomas of the rib	<30	26	Low initial suspicion of tumor (5/26), with fre-quent alternative diagno-ses (e.g., pleurisy in 11/26).<br><br>Diagnostic delay shorter for patients reporting fe-ver or a palpable mass at the first visit.<br><br>Imaging at first presenta-tion often non-diagnostic, with normal or inconclu-sive findings in the ma-jority of cases.<br><br>Tumor suspected in 4/15 patients on initial chest X-ray.	Initial presentation to GP (15/26) and ED (10/26). One case detected inci-dentally.	High

Widhe et al. †† [51]	2000	Retro-spective cohort study<br><br>Sweden	1983-1995	Popula-tion-based	National register, medical charts	Osteosar-comas and Ewing sarcomas	<30	149	Symptoms were fre-quently attributed to trauma (48%), potentially delaying further investi-gation.<br><br>Tendinitis was the most common misdiagnosis (28%).<br><br>Radiograph was per-formed at the first medi-cal visit in 67% of osteo-sarcoma and 60% of Ewing sarcoma patients. Of these, 9% and 43%, re-spectively, were misinter-preted as normal or in-conclusive.<br><br>Significant associations were observed between shorter diagnostic delay and presence of a palpa-ble mass, radiography at first visit, and reported trauma, while absence of these factors was associ-ated with longer delay.	Initial presentation to GP (58%), Emergency De-partment (28%) and other providers (e.g., school or military doctors)	Low
Wine-stone et al. [52]	2024	Retro-spective cohort study<br><br>USA	2001-2017	Single-center	National register, insur-ance claims and elec-tronic health record database	All ma-lignant bone tu-mors	<40	1,801	Not reported.	Initial presentation to pri-mary care (56%), non-on-cology specialists/sur-geons (17%), urgent care/inpatient presenta-tion (2%)	High

Yoshida et al. [57]	2021	Retro-spective cohort study<br><br>United Kingdom	2004-2018	Single-center	Medical charts	Osteosar-comas	4-16	250	Not reported.	Not reported.	Moder-ate
Younger et al. [54]	2018	Cross-sectional observa-tional study<br><br>England	2015	Popula-tion-based	National survey, patient question-naires	All bone sarcomas	18-39	23	Initial GP management was split between referral (10/20) and reassurance or treatment for other conditions (10/20).<br><br>Emergency department more likely to initiate in-vestigation compared to GP.<br><br>Low initial suspicion of sarcoma (2/22).	GP involvement in diag-nostic pathway in 20/22 patients and Emergency Department involvement in 9/22 patients.	High

* Findings are based on a study population that extends beyond the predefined age range of 0–39 years.

** No further specification was provided beyond the number of patients aged 0–15 years and those aged ≥15 years.

† Classified as a retrospective cohort study due to its use of previously recorded clinical data, although described by the authors as mixed-methods.

†† Possible overlap with another study based on partially overlapping data collection periods, study site and/or author groups.
