# Supplementary material for: Diagnostic Intervals in Children, Adolescents and Young Adults with Primary Bone Sarcoma: A Systematic Review
Source: Cancers (Basel). 2026 Aug 12;18(16):2590. doi: 10.3390/cancers18162590 (PMC13510990; doi:10.3390/cancers18162590)
Supplement: Supplementary file 1 [file cancers-18-02590-s001.zip › Supplementary Table S5.pdf]

Supplementary Table S5. Reported Intervals Among Selected Studies.

	Age groups	n	Intervals	Median (days)	Mean (days)	Range (days)	IQR (days)	Diagnosed (%)		Interval and time point definitions
								<1 month	>1 month	
Ahrens-berg et al. [33]	<15 years	19	Patient interval	19	…	…	0-28	…	…	The time from the first symptom related to the malignancy until the first presentation to the GP or another health care provider
			Diagnostic interval	45	…	…	11-60	…	…	The time from the first presentation until the date of diagnosis
			Total interval	88	…	…	57-132	…	…	The time span from symptom start to treatment start
Bacci et al. [34]	<20 years	607	Total diagnostic interval	…	72.1*	…	…	…	…	Interval between onset of symptoms and final diagnosis (time of diagnostic biopsy)
Berlanga et al. [35]	≤22 years	85	Total diagnostic interval	56*	…	0-700	…	…	…	Duration of symptoms prior to diagnosis (time of initial biopsy)
Brasme et al. [36]	<21 years	436	Total diagnostic interval	70	…	1-1,461	27-146	72	…	The time from onset of symptoms to the date of diagnosis
			Total interval	83	…	…	38-157	…	…	The interval between symptom onset and the first day of chemotherapy

Brotzmann et al. [37]	All ages	13	Total diagnostic interval	150**	319**	30-1,034**	…	…	…	The time period between the first clinical symptoms and the diagnosis (based on histology after biopsy)
Chen et al. [38]	<25 years	30	Total diagnostic interval	86.5	…	7-1,483	41-156	…	…	The time between symptom onset and diagnosis of a malignancy
Dang-Tan et al. [39]	<20 years	142	Patient interval	30	…	…	4-62	…	…	From first symptom to initial health care contact
		145	Diagnostic interval	15	…	…	6-37	…	…	From first medical contact to cancer diagnosis
		143	Total diagnostic interval	61	…	…	32-114	…	…	From cancer symptom detection to cancer diagnosis
		141	Treatment interval	7	…	…	4-12	…	…	From cancer diagnosis to start of first treatment
		142	Total interval	66	…	…	41-121	…	…	From cancer symptom detection to start of first treatment
Desandes et al. [40]	15-19 years	33	Total diagnostic interval	70.7*	…	…	40.6-191.8*	…	…	Interval between the onset of evidence of an existing malignancy (cancer symptom detection or sign) and the date of a verified cancer diagnosis
			Treatment interval	17	…	…	9-28	…	…	Interval between the date of cancer diagnosis and the starting date of treatment
	20-24 years	15	Total diagnostic interval	149.8*	…	…	21-378*	…	…	Interval between the onset of evidence of an existing malignancy (cancer symptom detection or sign) and the date of a verified cancer diagnosis

			Treatment interval	24	…	…	0-43	…	…	Interval between the date of cancer diagnosis and the starting date of treatment
Dommett et al. [53]	15-24 years	7	Patient interval	20	…	0-417	…	…	…	Time from first symptom to first presentation
			Diagnostic interval	81	…	24-140	…	…	…	Time from first presentation/clinical appearance to diagnosis
			Treatment interval	21	…	2-44	…	…	…	Time from diagnosis to start of treatment
			Total interval	86	…	45-169	…	…	…	Time from first presentation to start of treatment
Goyal et al. [41]	≤22 years	103	Total diagnostic interval	115.5**	…	30.4-1,398.4**	…	…	…	The time from first onset of symptoms or signs to a definitive diagnosis
Hajjaj et al. [42]	≤18 years	33	Treatment interval	11	…	…	6-16	94	6	The time from diagnostic biopsy to first treatment
Herbert et al. [22]	12-24 years	68	Total diagnostic interval	107	…	…	53-222	…	…	Time of symptom onset to cancer registry diagnosis date
			Patient interval	…	…	…	…	75	25	From symptom onset to first presentation to a GP or the emergency department
Kager et al. [43]	<5 years	24	Total diagnostic interval	28*	…	7-84*	…	…	…	Duration of symptoms before diagnostic biopsy
Lawrenz et al. [44]	18-30 years	3,220	Treatment interval	19	…	…	8-35	…	…	Interval between symptom onset to first presentation to the general practitioner or accident or emergency

Martin et al. [45]	15-29 years	29	Total diagnostic interval	…	110	…	…	…	…	The interval between the onset of evidence of an existing malignancy (symptom or sign) and the date on which a specific cancer type was verified by pathological confirmation
Martin et al. [56]	25-39 years	10	Patient interval	13	…	1-372	…	…	…	The time from the patient noticing the first symptom to the first consultation with a healthcare professional
			Diagnostic interval	123.5	…	10-799	…	…	…	The time between the patient's first encounter with a healthcare professional to receiving a cancer diagnosis
Nandra et al. [46]	<22 years	1,367	Patient interval	84	…	…	42-140	…	…	Duration of symptoms prior to first clinic attendance
Ruggieri et al. [47]	<15 years	27	Total diagnostic interval	…	…	60.8-182.4**	…	…	…	Interval between first symptoms and diagnosis
Shanmu-gadivel et al. [55]	0-18 år	124	Patient interval	21.7*	58.8*	0-1,148*	2.1-60.9*	…	…	Time between symptom onset and first presentation to healthcare
			Diagnostic interval	32.2*	77.7*	0-1,103.2*	13.3-84*	…	…	Time between first presentation and diagnosis
			Total diagnostic interval	88.2*	137.7*	0-1,171.1*	46.2-162.4*	…	…	Time between symptom onset and diagnosis
Veneroni et al. [48]	Pediatric population †	65	Total diagnostic interval	84	…	…	35-165	…	…	Time from the initial onset of symptoms to the final diagnosis
				30	…	…	5-80	…	…	

			Patient interval							Time from the initial presentation of symptoms to the first contact with a doctor
Venka-teswaran et al. [49]	<22 years	33	Total diagnostic interval	91.2**	…	30.4-729.6**	…	…	…	From onset of symptoms to diagnosis
Widhe et al. †† [50]	<30 years	26	Patient interval	76**	…	0-304**	…	…	…	The period from when symptoms were first noticed until the first visit to a doctor
			Diagnostic interval	91.2**	…	0-304**	…	…	…	The period from the first visit until the time that the diagnosis of Ewing sarcoma was established
Widhe et al. † [51]	<30 years‡	102	Patient interval	…	42*	7-182*	…	…	…	The period from when the patient first noted symptoms until the patient first consulted a doctor
			Diagnostic interval	…	63*	7-364*	…	…	…	The period from the first medical visit for these symptoms until the date that an accurate diagnosis was established
			Total diagnostic interval	…	105*	14-525*	…	…	…	The period from when the patient first noted symptoms until the date that an accurate diagnosis was established
	<30 years‡‡	47	Patient interval	…	105*	7-700*	…	…	…	The period from when the patient first noted symptoms until the patient first consulted a doctor
			Diagnostic interval	…	133*	7-504*	…	…	…	The period from the first medical visit for these symptoms until the date that an accurate diagnosis was established
				…	238*		…	…	…	

			Total diagnostic interval			21-1,050*				The period from when the patient first noted symptoms until the date that an accurate diagnosis was established
Wine-stone et al. [52]	<40 years	1,801	Total diagnostic interval	40	…	…	8-127	…	…	The time from first symptoms captured in the claims to definitive diagnosis
Younger et al. [54]	18-39 years	23	Patient interval	…	…	…	…	54.6	45.4	Time from first symptom to presentation to a medical professional

* Converted from weeks to days by multiplying by 7. ** Converted from months to days by multiplying by 30.4.
† No further specification was provided beyond the number of patients aged 0–15 years and those aged ≥15 years.
‡ Osteosarcoma patients. ‡‡ Ewing sarcoma patients.
