# Supplementary material for: Diagnostic Intervals in Children, Adolescents and Young Adults with Primary Bone Sarcoma: A Systematic Review
Source: Cancers (Basel). 2026 Aug 12;18(16):2590. doi: 10.3390/cancers18162590 (PMC13510990; doi:10.3390/cancers18162590)
Supplement: Supplementary file 1 [file cancers-18-02590-s001.zip › Supplementary Table S6.pdf]

**Supplementary Table S6. Risk of Bias Assessment across Included Studies.**

| Study (Author, year) | Population representativeness | Definition & measurement of intervals | Data completeness | Reporting transparency | Bias & confounding | Overall Risk of Bias |
|---|---|---|---|---|---|---|
| Ahrensberg et al. [33] | Low | Moderate | High | Low | Moderate | Moderate |
| Bacci et al. [34] | High | Moderate–high | High | High | High | High |
| Berlanga et al. [35] | Moderate | High | High | Moderate | Moderate–high | Moderate–high |
| Brasme et al. [36] | Low | Low | Moderate | Low | Low–moderate | Low |
| Brotzmann et al. [37] | High | High | Moderate–high | Moderate | High | High |
| Chen et al. [38] | Moderate | High | Moderate–high | Moderate | High | High |
| Dang-Tan et al. [39] | Low–moderate | Low | Low | Low | Low–moderate | Low |
| Desandes et al. [40] | Low | Moderate | Moderate | Low | Moderate | Moderate |
| Dommett et al. [53] | High | Moderate–high | High | Moderate | High | High |
| Ferrari et al. [60] | Moderate–high | Moderate | Moderate–high | Moderate | Moderate | Moderate |
| Goyal et al. [41] | Moderate | Moderate | Moderate | Low–moderate | High | Moderate |
| Hajjaj et al. [42] | Low | Moderate | Moderate | Moderate | Moderate | Moderate |
| Herbert et al. [22] | Moderate | Moderate–high | Moderate | Moderate | Moderate | Moderate–high |
| Kager et al. [43] | Moderate–high | High | Moderate | High | Moderate | High |
| Kell et al. [59] | Moderate–high | High | High | Moderate–high | Moderate | Moderate–high |

| | | | | | | |
|---|---|---|---|---|---|---|
| Kha et al. [58] | Moderate–high | Moderate | Moderate | Moderate | Moderate–high | Moderate |
| Lawrenz et al. [44] | Moderate | Low | Moderate | Moderate | Low–moderate | Low–moderate |
| Martin et al. [45] | Moderate–high | High | Moderate | Moderate | Moderate | Moderate |
| Martin et al. [56] | High | High | Moderate | Moderate–high | High | High |
| Nandra et al. [46] | Moderate–high | Moderate | High | Moderate–high | High | High |
| Ruggieri et al. [47] | High | High | High | High | High | High |
| Shanmugadivel et al. [55] | Low | Low | Low | Low | Low–moderate | Low |
| Veneroni et al. [48] | Moderate | Moderate | Low | Low to moderate | Moderate | Moderate |
| Venkateswaran et al. [49] | High | High | Moderate–high | Moderate | High | High |
| Widhe et al. †† [50] | Moderate–high | High | Moderate–high | Moderate | High | High |
| Widhe et al. † [51] | Low–moderate | High | Moderate | Moderate | High | Moderate |
| Winestone et al. [52] | Moderate | High | Moderate–high | Moderate | Moderate | Moderate |
| Yoshida et al [57] | Moderate–high | Moderate | Moderate–high | Moderate | Moderate | Moderate |
| Younger et al. [54] | High | High | Moderate | Low–moderate | High | High |
